# Timely Lessons From the Pandemic in Turkey

**DOI:** 10.1017/dmp.2020.376

**Published:** 2020-10-12

**Authors:** Hakan Yaman

**Affiliations:** Independent Scholar, Antalya, Turkey

**Keywords:** COVID-19, communicable diseases, disasters, disease outbreaks

The coronavirus disease (COVID-19) pandemic has affected all countries worldwide. Turkey was not an exception. Soon after the novel coronavirus was detected in Wuhan on January 10, 2020, the Turkish Ministry of Health (TMoH) established a scientific advisory board where experts of COVID-19 relevant medical fields were invited. The number of advisory board members increased gradually to 38 by adding new specialists.^1^ The guidelines of this board were amended by the TMoH, and the health care sector of Turkey used this regularly updated, country-specific document during their clinical services.^2^ Two weeks later, airports started to screen travelers to detect COVID-19 cases, and, by the end of January, Turkish citizens in Wuhan were evacuated. On February, flights from China and later from Iran, Italy, South Korea, and Iraq were stopped. The first case of COVID-19 in Turkey was reported on March 11.^3^ On March 16, all schools and universities were closed temporarily and sports events postponed. On March 15, 10 320 Turkish citizens who returned from Umrah (an Islamic pilgrimage to Mecca at any time of the year) and regardless of their test status for COVID-19, were quarantined in 3 student dormitories in Ankara (n = 5392) and 2 in Konya (n = 4938) for 14 days.^4^ Congregational prayers in mosques were also banned. On March 17, the first death from COVID-19 was confirmed.^5^ On March 20, hospitals were declared as pandemic hospitals.^6^ Later on, people over age 65 and under age 20 were prohibited to leave home temporarily. As every age group is at risk of infection, people who were not in need of active work were asked to stay at home. After the closure of schools, students from kindergarten to university were confined. Even the government provided financial aid to people in need, a certain part of population had to go to work, and most of them were in the age group of 20–64 years. These people were not restricted to leave home.

The COVID-19 Turkey Web Portal has been launched by the Turkish Scientific Research Institution and the Turkish Ministry of Industry and Technology, which supports research in vaccine and test development.^7^ Health workforces were forbidden to leave their posts for months. On April 29, 7428 health workers (6.5%) tested positive for COVID-19.^8^ Later, a 15-day entrance ban to 30 metropolitan cities and Zonguldak was declared. Around the end of May, the “new normal” of Turkey had been declared by the government.^9^ In June, domestic flights had begun, and public spaces opened for visitors. Preventive measures still continue today. Intercity travels and flights have reduced their seats sold and seats are spaced to allow for social distancing. Shops are accepting only customers wearing masks, and the number of visitors is restricted according to their space. Barbers are allowed to welcome only 1 customer for each session. People over age 65 and under age 20 are allowed to leave home occasionally. Schools are expected to open by the end of August 2020. The health system is “normalizing” as well. The pandemic has been lifted and quarantine wards are closed. The workforce has shifted from part-time to full-time work. Outpatient clinics are now accepting patients for medical services. By June 25, 3 135 424 tests had been performed, 193 115 patients had been confirmed with COVID-19, 165 706 recovered, and 5046 died.^10^ Candidates for testing, quarantine, or hospitalization were as follows^11,12^:

1. Suspicious Case
  - Having at least 1 COVID-19 symptom (fever, cough, dyspnea, sore throat, headache, myalgia, loss of taste or smell, or diarrhea) AND having no other clinical explanation for symptoms, or being in a high-risk area of disease and in close person-to-person contact with a sick person in the last 14 days before symptom onset.
    OR
  - Having at least 1 COVID-19 symptom (fever, cough, dyspnea, sore throat, headache, myalgia, loss of taste or smell, or diarrhea) AND having no other clinical explanation for symptoms or being in close contact with a patient diagnosed with COVID-19 in the last 14 days before symptom onset.
    OR
  - Having at least 1 COVID-19 symptom (fever, cough, dyspnea, sore throat, headache, myalgia, loss of taste or smell, or diarrhea) AND having no other clinical explanation for symptoms or having severe acute respiratory infection and respiratory symptoms onset in the last 14 days.
    OR
  - Having at least 1 COVID-19 symptom (fever, cough, dyspnea, sore throat, headache, myalgia, loss of taste or smell, or diarrhea) AND having no other clinical explanation for symptoms.
2. Definitive Case: Molecular Diagnosis of Severe Acute Respiratory Syndrome Coronavirus 2 (SARS-CoV-2)

Even the epidemic curve of Turkey is expected to decline to the lowest level in September, and the health authorities are alerted for new epidemic spikes in the coming weeks.

The curfew of citizens under age 21 and over age 64 seems to have a positive impact on COVID-19 pandemic management in Turkey. The lockdown of educational institutions, flexible working hours of governmental services, postponement of law court trials, and total lockdown of metropolitan areas on some weekends supported the positive outcome in Turkey.
